# Development and evaluation of one step single tube multiplex RT-PCR for rapid detection and typing of dengue viruses

**DOI:** 10.1186/1743-422X-5-20

**Published:** 2008-01-30

**Authors:** Parag Saxena, Paban Kumar Dash, SR Santhosh, Ambuj Shrivastava, Manmohan Parida, PV Lakshmana Rao

**Affiliations:** 1 Division of Virology, Defence Research & Development Establishment, Jhansi Road, Gwalior 474 002, MP, India

## Abstract

**Background:**

Dengue is emerging as a major public health concern in many parts of the world. The development of a one-step, single tube, rapid, and multiplex reverse transcription polymerase chain reaction (M-RT-PCR) for simultaneous detection and typing of dengue virus using serotype specific primers during acute phase of illness is reported.

**Results:**

An optimal assay condition with zero background was established having no cross-reaction with closely related members of flavivirus (Japanese encephalitis, West Nile, Yellow fever) and alphavirus (Chikungunya). The feasibility of M-RT-PCR assay for clinical diagnosis was validated with 620 acute phase dengue patient sera samples of recent epidemics in India. The comparative evaluation *vis a vis *conventional virus isolation revealed higher sensitivity. None of the forty healthy serum samples screened in the present study revealed any amplification, thereby establishing specificity of the reported assay for dengue virus only.

**Conclusion:**

These findings clearly suggested that M-RT-PCR assay reported in the present study is the rapid and cost-effective method for simultaneous detection as well as typing of the dengue virus in acute phase patient serum samples. Thus, the M-RT-PCR assay developed in this study will serve as a very useful tool for rapid diagnosis and typing of dengue infections in endemic areas.

## Background

Dengue is the most important mosquito borne viral infection and is prevalent in most parts of the tropics. Currently dengue fever causes more illness and death than any other arboviral disease in humans. An estimated 2.5 billion people live in the areas at risk for epidemic dengue virus transmission. One hundred million cases of dengue fever (DF) and 450,000 cases of dengue hemorrhagic fever/dengue shock syndrome (DHF/DSS) are reported annually [1-3]. The most challenging problem associated with dengue is patient management, which is possible through rapid diagnosis of early infection. In dengue infection, serotyping is very important because of the fact that secondary infection with a heterologous serotype often leads to life threatening dengue haemorrhagic fever (DHF) and dengue shock syndrome (DSS) [4].

Laboratory diagnosis of dengue infection is primarily achieved through virus isolation from acute phase serum, serodiagnosis and molecular assays for detection of viral RNA [5]. Virus isolation though considered 'gold standard' is technically demanding and time consuming. The most rapid serological technique, such as IgM ELISA with a single serum sample, does not furnish information about the serotype of the virus. The plaque reduction neutralization technique (PRNT) allows typing but is costly, slow and difficult to perform. The molecular methods based on PCR technique offer a suitable alternative to conventional isolation technique. The RT-PCR targeting the conserved regions of C-prM gene junction is widely employed for precise confirmation of an infection [6]. In routine practice, a two-step method with a RT-PCR followed by a nested PCR is used for serotyping of dengue viruses. However, this method is expensive, time consuming, and also suffers from carryover contamination problems. To improve on this, we report here the development of a one-step single-tube rapid multiplex PCR assay for rapid detection and differentiation of dengue serotypes in acute phase serum samples.

## Results

**Figure 1:**
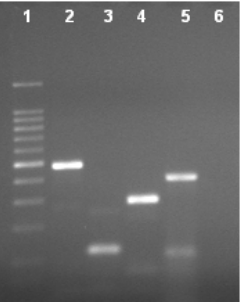
1.5% Agarose gel electrophoresis demonstrating the correctly sized amplicons generated by the single tube dengue multiplex RT-PCR. Lane 1: 100 bp DNA ladder (Fermentas), lanes 2–6: Dengue 1 (482 bp), Dengue 2 (119 bp), Dengue 3 (290 bp), Dengue 4 (389 bp) and negative control.

All the viruses were propagated in C6/36 cells prior to extraction of RNA. The isolation of virus on C6/36 cells led to successful isolation of Dengue type 2, 3 and 4 viruses (Table 1). RNA was extracted from culture supernatant and used as template in RT-PCR and nested PCR. The RT-PCR and nested PCR revealed desired amplification as reported earlier [6]. The multiplex PCR was standardized as a single tube method by incorporating all the four-serotype specific primers, along with a dengue consensus forward primer. The assay was standardized by optimizing the annealing temperature (55°C), primer (20pmol) concentration that resulted in generation of differently sized serotype specific amplicons viz., Dengue 1 (482 bp), Dengue 2 (119 bp), Dengue 3 (290 bp), and Dengue 4 (389 bp) as shown in the Figure 1. Following standardization, the assay was further optimized for screening RNA extracted from clinical samples.

**Table 1:** Distribution of serotypes among confirmed dengue positive cases by multiplex RT-PCR (M-RTPCR)

**Dengue virus serotypes**	**Number of patients found infected with dengue viruses (serotype-wise distribution)**	
	
	**M-RTPCR (*n *= 96)**	**Cell culture supernatant (*n *= 57)**
**DEN-1**	2	0
**DEN-2**	47	31
**DEN-3**	42	25
**DEN-4**	5	1
**Total**	96	57

### Sensitivity and Specificity of multiplex RT-PCR

The detection limit of both dengue complex and the Multiplex RT-PCR assay was determined through 10 fold serial dilution of RNA copies. The RNA copies were generated using *in vitro *transcription. The sensitivity of dengue complex and multiplex RT-PCR was found to be 500 and 2500 RNA copies respectively. The multiplex PCR was also compared with gold standard virus isolation and the sensitivity was found to be much higher (96 compared to 57 in isolation) (Table 2). The serotypes of all these isolates were confirmed using both multiplex RT-PCR and two step RT-PCR followed by nested PCR, which revealed 100% concordance (Details not shown). The isolation results was further confirmed by sequencing of the respective amplicons, which revealed perfect matching with the respective serotype specific sequences, confirming the specificity of this assay (data not shown). The specificity of the multiplex RT-PCR assay was compared with closely related flavi- and alphaviruses. It was observed that this assay is highly specific for dengue serotypes with no cross reactivity with Japanese encephalitis, West Nile, Yellow fever and Chikungunya viruses. Furthermore, all the serum samples from a panel of 40 healthy individuals analyzed in this study revealed no amplification, thereby establishing the specificity of this assay.

**Table 2:** Comparison of Multiplex PCR and Virus isolation for the detection and serotyping of dengue viruses from acute phase serum samples (n = 620)

	**M-RT-PCR**	**Virus isolation**
**Positive**	96 (15.48%)	57 (9.20%)
**Negative**	524 (84.52%)	563 (90.80%)

### Evaluation of Multiplex RT-PCR

The feasibility of the assay for clinical diagnosis was validated by evaluating with serum samples from 620 acute-phase suspected patients and 40 healthy individuals from the same area. On comparative evaluation with RT-PCR, the multiplex RT-PCR was found to be equally sensitive for detection of dengue viral RNA in patient sera.

## Discussion

Dengue cases are increasing over the years in many parts of the world. Now it is endemic in India, with circulation of multiple serotypes [7,8]. There is also an increased incidence of fatal DHF and DSS, which requires urgent medical intervention [9]. Early diagnosis of dengue is critical in the absence of any licensed antiviral therapy and prophylaxis. The diagnosis is achieved either serologically by detecting dengue-specific IgM and IgG antibodies, which generally appear 7–8 days after the onset of illness [10]. However, it is of less value for early diagnosis. In addition, persistent circulation of IgM antibodies for more than 90 days also is a limiting factor in confirmatory diagnosis. The detection of IgG is generally not considered authentic, due to cross reactivity with other closely related members of Flaviviruses. This needs to be confirmed with paired sera, which is not practical in most cases [11,12].

The detection of dengue serotype is very important due to fact that in secondary cases, infection with a heterologous serotype often leads to fatal DHF and DSS [13]. So, early typing is a pre-requisite for proper patient management. A large number of molecular methods have been reported for serotyping of dengue viruses [6,14-18]. However, most of these methods are four tube based RT-PCR followed by nested PCR or four serotype specific RT-PCR. The multiplex PCR assay described by Harris et al., (1998) [16] also suffers from lack of proper evaluation with clinical samples. To improve on the existing assay systems, we standardized a one step single tube protocol for rapid serotyping of dengue viruses. This assay can be performed rapidly with in a period of 4 hours compared to 8 hours in two-step typing assays. This can provide faster information to the clinicians leading to initiation of suitable symptomatic therapy. This single tube protocol also reduces the cost by four fold, resulting in an economical way of serotyping [16]. The two-step assays are always more prone to contamination due to opening of tubes between the steps. All these advantages make this assay a user friendly, rapid, cost effective diagnostic tool and can be utilized in many developing countries, where dengue is endemic.

The sensitivity and specificity of the single-tube multiplex RT-PCR was also verified. It could correctly serotype all the four respective dengue serotypes. No cross-reaction was observed when other related flaviviruses and alpha viruses were included. The processing of all the 96 PCR positive samples for virus isolation, led to 57 dengue isolates. This isolation was confirmed by both nested PCR and multiplex PCR, which revealed similar results. This indicates the lower sensitivity of isolation compared to RT-PCR, which may be attributed to either inactivation of virus during transportation or failure in maintenance of cold chain [7]. The sensitivity was further checked by developing RNA transcripts. The detection limit of multiplex PCR was found to be 2500 copies. Though it is 5 fold less sensitive compared to group specific RT-PCR, however, its importance can be judged from the fact that this assay gives precise information regarding the serotype of the virus.

## Conclusion

The development of a suitable effective vaccine and therapeutics for dengue is not yet achieved and it is presumed that it will not be available for coming few years. In these circumstances, rapid diagnosis can help in timely patient management. The multiplex PCR assay developed in this study will be extremely useful for rapid diagnosis and serotyping of viruses in dengue infections.

## Methods

### Virus

Virus strains of the four standard dengue serotypes were obtained from the National Institute of Virology, Pune, India (Den-1, Hawaii; Den-2, P-23085; Den-3, 633798 and Den-4, 642069). The dengue viruses isolated during this study from dengue outbreak in India since 2001 were also included. To check the cross reactivity, related flaviviruses viz., Japanese encephalitis (JaOArS982), West Nile (G22886), Yellow fever (17D vaccine strain) virus and one alphavirus (Chikungunya (S27) were also included in this study. These viruses were propagated in C6/36 cells.

### Clinical Samples

A total of 620 human serum samples from febrile patients clinically suspected of having dengue fever were collected within 0 to 4 days from the time of onset of symptoms. These samples were collected from different parts of India during various outbreaks from 2001–2007 [7,8]. In addition, a panel of 40 serum samples collected from healthy volunteers from the same area were included as negative control in this study.

### Virus isolation

C6/36 cells [19] were grown in Eagle's minimum essential medium (EMEM) (Sigma, USA) supplemented with 10% Tryptose Phosphate Broth (TPB) (DIFCO, USA), 10% Fetal bovine serum (FBS) (Sigma, USA), 3% L-glutamine (Sigma, USA) and gentamicin (80 mg/l) (Nicholas-Piramal, India) at 28°C. Isolation of viruses from acute phase dengue suspected samples were attempted following the standard virus adsorption technique [20]. Briefly, preformed manolayer of cells were washed with plain medium prior to infection. The virus/suspected serum was allowed to adsorb to the cells for 1 hr at 37°C. Following adsorption, the inoculum was replenished with 2 ml of maintenance medium (EMEM with 2% FBS). Suitable cell controls were also kept along side. The cells were harvested on appearance of cytopathic effects or on 6^th ^day post inoculation (dpi), whichever is earlier. The identification of the virus isolates obtained from the clinical samples was carried out by RT-PCR as described below.

### RNA Extraction

RNA was extracted from standard viruses, virus isolates, sera of suspected dengue patients and healthy volunteers using QIAquick viral RNA mini Kit, following the manufacturer's protocol. Finally, the RNA was eluted in 60 μl of diethyl pyrocarbonate (DEPC) treated water (Sigma, USA).

### Dengue complex Reverse transcription polymerase chain reaction (RT-PCR)

Conventional Dengue complex RT-PCR assays were performed according to the protocol [6] with slight modifications. Briefly, the RT-PCR was performed with RNA from standard dengue viruses and confirmed dengue virus-infected patient serum samples initially in a 50 ul reaction volume using Access quick RT-PCR kit (Promega, USA) with dengue virus group-specific consensus primers (D1: 5' TCAATATGCTAAAACGCGCGAGAAACCG 3' and D2: 5' TTGCACCAACAGTCAATGTCTTCAGGTTC 3').

### Dengue Nested PCR

The nested PCR assay was performed according to the protocol [6] with slight modifications. Briefly, the 1: 10 dilution of RT-PCR amplicon was used as template in the nested PCR in a 50 μl reaction volume using master mix of Access quick RT-PCR kit, with dengue virus group-specific consensus forward primer (D1), and four serotype specific reverse primers (Ts1: 5' CGTCTCAGTGATCCGGGGG 3', Ts2: 5'CGCCACAAGGGCCATGAACAG 3', Ts3: 5' TAACATCATCATGAGACAGAGC 3' and Ts4: 5'TGTTGTCTTAAACAAGAGAGGTC3'), as reported earlier [6].

### Single-step Dengue multiplex RT-PCR (M-RT-PCR)

A one-step single tube serotype-specific multiplex PCR was performed with RNA from standard dengue viruses and confirmed dengue virus-infected patient serum samples using a multiplex RT-PCR protocol. The amplification was carried out in a 50 μl total reaction volume with Access quick RT-PCR kit according to the manufacturer's protocol, along with five primers viz., forward D1 and four serotype specific reverse primers (Ts1, Ts2, Ts3 and Ts4).

### Evaluation of multiplex RT-PCR

The evaluation of the multiplex RT-PCR assay was carried out with 620 serum samples collected over a period of six years from India.

### Preparation of RNA standard

The detection limit of this assay was determined using RNA standards. The RNA standard was produced using T7 transcription kit (MBI Fermentas, USA) following the manufacturer's protocol. Initially PCR amplicons of respective dengue serotypes were generated using a modified D1 primer (T7 promoter sequence (TAATACGACTCACTATAGG) was added at the 5' end of D1 primer) and normal D2 primer. These amplicons (530 bp) of all the four serotypes were gel purified, quantitated, before being used as template in transcription reaction. The purified template was subjected to in vitro transcription (IVT) at 37°C for 1 h. The IVT products were then treated with 1 U of DNase I and incubated at 37°C for 15 min to remove the remaining DNA followed by inactivation of DNase I at 70°C for 15 min. The IVT products were ethanol precipitated and resuspended in DEPC treated water. The amount of respective dengue serotype specific RNA transcripts were determined spectrophotometrically and converted to molecular copies by using the following formula 21.

*Y *(molecules/μl) = [*X*(g/μl)/transcript length (nucleotides) × 340] × 6.023 × 10^23^

### Sensitivity and specificity of the assay

The sensitivity of both the dengue complex and serotype specific multiplex RT-PCR assay was determined through serial dilutions of the RNA transcripts. The specificity was determined on comparison with related Flaviviruses (JE, West Nile and Yellow fever viruses), alphavirus (Chikungunya virus) and a panel of 40 serum samples from healthy volunteers.

## Competing interests

The author(s) declare that they have no competing interests.

## Authors' contributions

PS carried out the standardization of all RT-PCR experiments, virus isolation, and evaluation of the M-RT-PCR assay. PKD carried out the nested PCR and *In vitro *transcription and sequencing. SSR carried out the RT-PCR, IVT assay. AS carried out the virus isolation and evaluation with clinical samples. MMP conceived the study and planned the work. PVLR helped out to design and draft the manuscript and also revised it critically. All authors read and approved the final manuscript.
